# A Comparison of Safety and Effectiveness Between Wingspan and Neuroform Stents in Patients With Middle Cerebral Artery Stenosis

**DOI:** 10.3389/fneur.2021.527541

**Published:** 2021-05-20

**Authors:** Kai Zhou, Yuan Cao, Xiao-Hui He, Zhong-Ming Qiu, Shuai Liu, Zi-Li Gong, Jie Shuai, Qing-Wu Yang

**Affiliations:** Department of Neurology, Xinqiao Hospital, Army Medical University, Chongqing, China

**Keywords:** Neuroform, Wingspan, in-stent restenosis, complications, intracranial artery stenosis

## Abstract

**Background:** Percutaneous transluminal angioplasty and stenting with the Wingspan stent has proven safe and effective in patients with middle cerebral artery stenosis (MCAS), but the off-label use of the Neuroform stent might be an alternative treatment. This study aimed to compare the safety and effectiveness of the above two intracranial stents in patients with MCAS.

**Methods:** We retrospectively analyzed consecutive patients with symptomatic MCAS who had been treated with the Neuroform EZ or the Wingspan stent. A propensity score was generated to control for differences in baseline characteristics. The endpoints were the rate of peri-procedural complications within 30 days after stenting, the in-stent restenosis rate, and any target-vessel-related stroke or deaths during follow-up.

**Results:** After matching for propensity score, the peri-procedural complication rate in the Wingspan group was 7.4% compared with 5.6% in the Neuroform group (*p* = 1.00), while the follow-up in-stent restenosis rates were 23.3 vs. 14.3%, respectively (*p* = 0.41). In the restenosis group, the patients tended to be younger (*p* < 0.01) and the degree of artery stenosis before stenting was higher (*p* < 0.01).

**Conclusion:** This study indicated that in patients with symptomatic MCAS, Neuroform EZ stents are an alternative to Wingspan. Moreover, younger age and higher degree of artery stenosis before stenting might be a risk factor of in-stent restenosis.

## Introduction

In the last decade, studies have revealed that intracranial large-artery stenosis or occlusion (ICAS) is more common in patients of Asian, Black, and Hispanic ancestry than in patients who are white (1, 2), and that the incidence of stroke recurrence is much greater with ICAS than without (15–22.1 vs. 5.3–5.5%, *p* < 0.01) (3, 4). Thus, aggressive therapeutic strategies, such as dual antiplatelet treatment or percutaneous transluminal angioplasty and stenting (PTAS) have been adopted to reduce stroke recurrence in these patients. Subgroup analysis of patients in the CHANCE study revealed that in patients with ICAS, the recurrence rate of stroke had almost the same high value in the mono-antiplatelet group as in the dual-antiplatelet group (13.6 vs. 11.3%, *p* > 0.05) (5). This failure of the best medical therapy for secondary prevention of stroke makes the PTAS approach a tantalizing prospect for neural interventional groups around the world aiming to reduce the recurrence of stroke in the territory of the stenotic artery.

Unfortunately, two randomized controlled studies have shown that stenting plus the best medical therapy was not superior to the best medical therapy alone because of the high incidence of peri-operative ischemic or hemorrhagic stroke events (6, 7). Follow-on studies resorted to improved patient and device selection methods to bring down the peri-operative event rate (8). However, long-term follow-up data were not obtained. Therefore, we cannot tell from these results whether stenting plus best medical therapy is superior to best medical therapy alone in stroke prevention. The safety of stenting has nevertheless been verified by us and other researchers (9–12). Wingspan (Stryker Neurovascular, Fremont, CA, USA), the only stent approved for intracranial atherosclerotic stenoses, was widely used in the studies cited above, but other retrospective studies have indicated that stenting with Enterprise^TM^ (Cerenovus, Fremont, CA, USA) is also safe and effective in patients with symptomatic ICAS (13–15). Comparing the technical parameters of Wingspan with those of the other intracranial stents, Wingspan exerts the highest radial force, which results in the highest in-stent restenosis (ISR) rate among the stents considered (16). These studies implied that other intracranial stents besides Wingspan may also be safe and effective and even yield a lower rate of ISR.

The Neuroform stent was first adopted for the process of aneurysm coiling. The stent is released after deployment of the ancillary microcatheter in the target stenosis artery, making the delivery of the stent to tortuous vessels much easier than with Wingspan (17). Given the lower radial force compared with Wingspan, the Neuroform stent can be deployed in small vessels with a diameter of less than 2 mm and the ISR rate is much lower than with other intracranial stents (18, 19). Giving the higher periprocedural and long-term symptomatic stroke rate in the treatment of perforator-bearing arteries in the posterior circulation (20), in the present study, we aimed to compare the safety and effectiveness of Neuroform EZ and Wingspan stents in patients with symptomatic middle cerebral artery (MCA) stenosis.

## Methods

### Patients

We retrospectively analyzed consecutive patients with symptomatic MCA stenosis who had been treated with either Neuroform or Wingspan from 1 January, 2018 to 1 January, 2021, using the stroke database of the Department of Neurology at the Second Affiliated Hospital of the Third Military Medical University.

The inclusion criteria were as follows: (1) recurrence of transient ischemic attack (TIA) or ischemic stroke (NIHSS ≤ 12; National Institutes of Health Stroke Scale) within the previous 180 days despite antiplatelet therapy; (2) digital subtraction angiography-verified severe atherosclerotic stenosis (≥70%) or occlusion in the M1 segments of the middle cerebral artery (MCA), using the method of the North American Symptomatic Carotid Endarterectomy Trial (NASCET) or (if the distal vessel was not accessible) the WASID measurement; (3) at least one atherosclerotic risk factor (hypertension, diabetes mellitus, hyperlipidemia, hyper-homocysteinemia, and smoking); (4) the previous stroke occurring more than 7 days before stenting; (5) age between 18 and 85 y; and (6) a drop in cerebral blood flow by 30% or more compared to the contralateral MCA circulation territory on computed tomography perfusion (CTP) imaging.

The exclusion criteria were as follows: a non-atherosclerotic lesion confirmed by high-resolution magnetic resonance imaging (HR-MRI); two or more stents placed after percutaneous transluminal angioplasty; concurrent intracranial pathology including tumors, aneurysms, or arteriovenous malformation; contraindication to heparin, aspirin, clopidogrel, or anesthetic; presence of metal implants contraindicated MRI; women during gestation; and life expectancy of <1 y due to other medical conditions.

The study was approved by the Medical Ethics Committee of Xinqiao Hospital, Army Medical University. Written informed consent was obtained from all participants or from an authorized family member. The study protocol was performed in accordance with relevant ethical guidelines and regulations for human studies.

### Preoperative Preparation

We followed our previously published methods of preoperative preparation (10). Briefly, coronary, and cranial computed tomographic angiography, brain CTP imaging, and the HR-MRI were obtained before stenting. Routine oral aspirin (100 mg/day) and clopidogrel (75 mg/day) were administered 3–5 days before stenting. The risk factors hypertension, low density lipoprotein, homocysteine, and high blood glucose were controlled according published guidelines for the prevention of secondary ischemic stroke (21). Smoking and drinking were prohibited, and each patient's lifestyle was adjusted. Patients with indications of coronary artery involvement were evaluated by cardiologists, and if there were no contraindications, implantation of coronary artery and intracranial artery stents were performed simultaneously.

### Stenting Procedure

Three experienced neurointerventionist with more than 100 cases of intracranial stent implantation performed the stenting procedure. After general anesthesia, the patients received 3,000 U of unfractionated heparin intravenously for systemic heparinization. Femoral artery puncture was performed using the Seldinger technique to place a 6F/8F arterial sheath. A 6F guiding catheter Envoy (Cerenovus, Fremont, CA, USA) was delivered to the C2–3 segment of the internal carotid artery. Over a Synchro-10 guidewire (Stryker Neurovascular, Fremont, CA, USA), an Echelon 10 microcatheter (Medtronic Neurovascular, CA, USA) was navigated to the distal part of the stenosis. The Synchro-10 guidewire was then retrieved, and through the microcatheter, an Exchangeable Synchro-14 guidewire (Stryker Neurovascular, Fremont, CA, USA) was advanced into the artery distal to the stenosis. If the path was too tortuous to deliver the microcatheter to the target lesion, the intermediate catheter Navien^TM^ (Medtronic Neurovascular, CA, USA) or a distal access catheter CAT^TM^ 5 (Stryker Neurovascular, Fremont, CA, USA) was passed coaxially through an 8F guiding catheter and was delivered to the C3–4 segment of the internal carotid artery to provide solid support of the system. Then, a Gateway balloon (Boston Scientific, Natick, MA, USA) was placed across the stenotic segment for balloon dilatation. Under-dilation was applied to avoid arterial dissection, vessel rupture, and snowplow effect of compressed plaque into perforator arteries (22, 23). The choice of stent type was decided by the neurointerventionists performing the procedure. The diameter of the target artery and the site of the lesion were the important factors, The Neuroform EZ (Stryker Neurovascular, Fremont, CA, USA) was preferred to Wingspan if the target artery was between 2 and 2.5 mm in diameter, or the target lesion was at the distal or bifurcate of the M1 segment of MCA, or the target lesion with tortuous anatomy. The selected Wingspan stent was placed in the lesioned vessel after balloon dilatation. If the Neuroform EZ stent was selected, the XT-27 microcatheter (Stryker Neurovascular, Fremont, CA, USA) was placed at least 10 mm distal to the stenosis site over the Exchangeable Synchro-14 guidewire after balloon dilatation, and then the Neuroform EZ stent was passed through the XT-27 coaxially to the stenosis locus. Finally, the stent was deployed by retreating the XT-27. After observation for 15 min, intracranial angiography was repeated. Repeated angioplasty was not performed after stenting. If no abnormalities were observed, the stenting procedure was completed. Successful revascularization was defined as follow: 1. the stent was successfully placed, 2. the residual stenosis was <50% both in the stent and at the edge of the stent in the range of 3 mm, 3. both acute in-stent thrombosis and distal arterial embolization were not visible in the following angiography after stenting.

### Peri-Operative Management

CT scanning (GE, Boston, MA, USA) was performed immediately after surgery to exclude brain hemorrhage. Blood pressure was controlled at 110–130/70–80 mmHg using antihypertensive drugs to prevent hyper-perfusion syndrome. If patients were free of hemorrhage, Enoxaparin Sodium (Sanofi Winthrop Industrie, France) was administered by subcutaneous injection at 4,000U/12 h for 3–5 days. Oral treatment with aspirin (100 mg/day) and clopidogrel (75 mg/day) was continued for at least 3 months, followed by long-term oral administration of either aspirin (100 mg/day) or clopidogrel (75 mg/day). Oral atorvastatin (40 mg/day) and probucol (0.5 g, twice daily) were also administered for at least 6 months following surgery.

### Evaluation of Endpoint Events

The follow-up modified Rankin Scale score (MRS) at the last clinical visit was obtained from the records in our stroke database. If the MRS was not available from our database, a telephone interview was initiated by a trained neurologist to bring the follow-up MRS data up to date as of 15 March, 2021. Follow-up computed tomography angiography (CTA) was scheduled at 1, 3, 6, or 12 months after surgery on a voluntary basis or when restenosis was suspected clinically. According the CTA results, ISR is defined as ≥50% of the stenosis occurring in the stent or within 3 mm of the edge of the stent.

The endpoints were: peri-procedural complications (death, ischemic stroke, or hemorrhagic stroke) within 30 days after the revascularization procedure, the rate of stroke recurrence in the area of the stented artery, and the incidence of ISR during the follow-up period. Ischemic stroke was defined as a ≥3 increase in the NIHSS score or a new focal neurologic deficit lasting ≥24 h not associated with a hemorrhage on brain CT. Hemorrhagic stroke was defined as a new neurological deficit or symptom or a ≥3 increase in the NIHSS score caused by parenchymal hemorrhage, subarachnoid hemorrhage, or intra-ventricular hemorrhage.

### Statistical Methods

Continuous data are presented as the mean ± standard deviation if normally distributed or as medians and interquartile ranges otherwise. Count data were examined using the χ^2^-test. The threshold for statistical significance was *p* < 0.05. For propensity score matching analysis, we performed a 1:1 matching based on the nearest-neighbor matching algorithm with a caliper width of 0.2 of the propensity score with gender, baseline NIHSS, Mori classification, and stenosis rate prior to stenting as covariates. For categorical variables, the intergroup differences were tested with the McNemar test and for continuous variables, with the paired *t*-test or the Wilcoxon matched-pairs signed rank test as appropriate. Statistical analyses were conducted using Graphpad Prism 6.0 or SPSS 21.

## Results

### Baseline Characteristics

Of the 190 included patents, 113 were assigned to the Wingspan group and 77 were assigned to the Neuroform group. The baseline characteristics of the patients showed more males in the Wingspan group (79.6 vs. 66.2%, *p* = 0.04). Meanwhile, higher NIHSS scores on admission (2.88 ± 3.19 vs. 4.69 ± 3.74, *p* < 0.01), higher degrees of stenosis in the target artery (84.59 ± 8.60 vs. 90.74 ± 10.78, *p* < 0.01), and more type-C in the Mori classification (34.5 vs. 66.2%, *p* < 0.01) were found in the Neuroform group. To eliminate the above differences, propensity scores were generated for the covariates gender, NIHSS, Mori classification, and stenosis rate prior to stenting using log odds. Matching generated 54 pairs of patients. The baseline characteristics of the matched pairs were compared and no significant difference were found (Table 1).

**Table 1:** Baseline characteristic of the patients.

**Characteristic**	**All patients included**		***p***	**Propensity score–matched patients**		***p***
	**Wingspan (*n* = 113)**	**Neuroform (*n* = 77)**		**Wingspan (*n* = 54)**	**Neuroform (*n* = 54)**	
Age	57.85 ± 10.69	61.65 ± 9.65	0.01^a^	58.19 ± 10.37	61.33 ± 9.54	0.10^c^
Male	90 (79.6%)	51 (66.2%)	0.04^b^	38 (70.4%)	42 (77.8%)	0.51^d^
NIHSS	2.88 ± 3.19	4.69 ± 3.74	<0.01^a^	3.63 ± 3.59	3.37 ± 3.33	0.70^c^
Hypertension	74 (65.5%)	58 (75.3%)	0.20^b^	37 (68.5%)	43 (79.6%)	0.27^d^
Diabetes	29 (25.7%)	26 (33.8%)	0.26^b^	12 (22.2%)	17 (31.5%)	0.39^d^
Hypercholesterolemia	24 (21.2%)	23 (29.9%)	0.23^b^	12 (22.2%)	16 (29.6%)	0.51^d^
Smoking	67 (59.3%)	37 (48.1%)	0.14^b^	32 (59.3%)	33 (61.1%)	1.00^d^
Homocysteine	13.28 ± 8.33	14.80 ± 15.36	0.51^a^	13.82 ± 5.56	14.14 ± 7.78	0.83^c^
Days from stroke to stenting	32.97 ± 34.77	24.57 ± 21.98	0.06^a^	31.85 ± 33.41	25.76 ± 22.79	0.27^c^
Mori classification			<0.01^b^			0.28^e^
Type A	23 (20.4%)	5 (6.5%)		3 (5.6%)	4 (7.4%)	
Type B	51 (45.1%)	21 (27.3%)		24 (44.4%)	16 (29.6%)	
Type C	39 (34.5%)	51 (66.2%)		27 (50.0%)	34 (63.0%)	
SR before stenting	84.59 ± 8.60	90.74 ± 10.78	<0.01^a^	86.57 ± 8.35	87.48 ± 11.21	0.63^c^
SR after stenting	13.32 ± 8.62	14.81 ± 11.37	0.31^a^	12.41 ± 8.17	13.70 ± 11.46	0.50^c^

*Baseline characteristics of the two groups before matched were compared with the use of either an independent group t-test(a) or a chi-square test(b). After matched, they were compared with the use of paired sample t-test(c), a McNemar test(d) or a Wilcoxon matched pairs signed rank test(e). NIHSS denotes national institute of health stroke scale; SR denotes stenosis rate*.

### Peri-Procedural Complications

We detected no significant between-group differences in the incidence of peri-procedural complications (5.3% in Wingspan group vs. 6.5% in Neuroform EZ group, *p* = 0.76) or lethal or disabling complications (1.7% in Wingspan group vs. 2.6% in Neuroform EZ group, *p* = 1.00). After matching, the incidence of peri-procedural complications also did not differ significantly between groups (7.4 vs. 5.6%, *p* = 1.00). Specifically, in the Wingspan group, 1 patient ultimately died of intracerebral hemorrhage, 1 patient experienced infarction in the perforating branch territory of the stented artery confirmed by MRI while the NIHSS returned to the baseline level when discharged, 1 patient had TIA, 2 had asymptomatic hemorrhagic transformation after infarction and 1 had symptomatic hemorrhagic transformation resulting from hyper-reperfusion. In the Neuroform group, no patient died during the peri-operative period, 1 patient had TIA, 1 patient experienced infarction the branch territory, 1 patient experienced guidewire perforation during the retreating of the delivery system (the patient was asymptomatic because the ruptured artery was successfully embolized by coil immediately), 1 patient experienced asymptomatic intracerebral hemorrhage, and 1 patient experienced symptomatic intracerebral hemorrhage (Table 2).

**Table 2:** Peri-procedural complications.

**Peri-procedural complications**	**All patients**		***p***	**Propensity score–matched patients**		***p***
	**Wingspan**	**Neuroform**		**Wingspan**	**Neuroform**	
	**(*n* = 113)**	**(*n* = 77)**		**(*n* = 54)**	**(*n* = 54)**	
Total	6 (5.3%)	5 (6.5%)	0.76	4 (7.4%)	3 (5.6%)	1.00
Transient ischemic attack	1	1		1	0	
Infarction	1	1		0	1	
Symptomatic ICH	2	1		2	1	
Asymptomatic ICH	2	2		1	1	
Lethal or disabled complications	2 (1.7%)	2 (2.6%)	0.70	1 (1.9%)	2 (3.7%)	0.30

*The incidence of total complications and lethal or disabled complications between the two groups were compared with the use of fisher's exact test*.

In order to analyze whether the perioperative complication was related to the time of stenting, “the early stenting group” included the patients whom underwent stenting within 14 days from stroke onset, “the delayed stenting group” included the patients whom underwent stenting > 14 days from last stroke occurred. The peri-operative complications occurred in 11.7% (6/51) of patients in early stenting group, compared to 3.6% (5/139) in delayed stenting group (*p* = 0.03). After matching, the peri-operative complications occurred in 9.7% (3/31) in acute phase vs. 7.8% (6/77) in chronic phase (*p* = 0.75).

### Events in Follow-Up and Prognosis

The binary MRS of 190 patients were obtained clinically or by telephone interview, and 5 patients were lost during the follow-up period. In the Wingspan group, one patient died of intracerebral hemorrhage, two patients experienced TIA or ischemic stroke in the territory of the stented artery after 30 days in the Wingspan group. In the Neuroform group, two TIA events occurred in the area of the target vessel and two patients experienced ischemic stroke during follow-up. The number of patients with poor prognosis were not significantly different between the two groups (3.6 vs. 6.7%, *p* = 0.49). After matching, we likewise detected no significant difference in numbers of patients with poor prognosis between the Wingspan and Neuroform groups (3.8 vs. 5.7%, *p* = 0.65).

The CT angiography was completed by us in a total of 152 patients during follow-up. ISR occurred in 19.7% of all patients included. The restenosis rate was 20.2% with a mean follow-up period of 8.3 months in the Wingspan group, while the restenosis rate was 19.0% with a mean follow-up period of 8.9 months in the Neuroform group. We observed no significant difference in the rate of restenosis between the two groups (*p* = 0.39). In the matched 102 patients, only 85 patients finished follow-up CT angiography. The rate of ISR was 18.8% with a mean follow-up period of 8.8 months. ISR was detected in 10 patients in the Wingspan group with a mean follow-up period of 8.7 months and in 6 patients in the Neuroform group with a mean follow-up period of 8.9 months. The rate of ISR did not differ significantly between the Wingspan and Neuroform groups (23.3 vs. 14.3%, *p* = 0.41). The baseline characteristics of all the patients and matched patients with follow-up CTA were listed in Table 3. The matched patients were further analyzed by binary logistic regression. The results indicated that older age might be a protective factor for restenosis (OR = 0.89, 95% CI 0.82–0.97, *p* < 0.01) and the stenosis rate might be a risk factor for restenosis (OR = 1.18, 95% CI 1.07–1.31, *p* < 0.01).

**Table 3:** Comparing patients with and without restenosis.

**Characteristic**	**All patients with follow-up CTA**		***p***	**Propensity score–matched patients with follow-up CTA**		***p***
	**Restenosis**	**Non-restenosis**		**Restenosis**	**Non-restenosis**	
	**(*n* = 30)**	**(*n* = 122)**		**(*n* = 16)**	**(*n* = 69)**	
Age	50.77 ± 8.11	60.86 ± 10.29	<0.01	51.63 ± 8.79	60.74 ± 9.72	<0.01
Gender (male, %)	23 (76.7%)	89 (73.0%)	0.68	14 (87.5%)	48 (69.6%)	0.22
Stent type (Wingspan, %)	18 (60.0%)	71 (58.2%)	1.00	10 (62.5%)	33 (47.8%)	0.41
NIHSS	4.33 ± 4.18	3.43 ± 3.29	0.21	5.25 ± 4.28	2.96 ± 3.00	0.01
Hypertension	18 (60.0%)	85 (69.7%)	0.38	10 (62.5%)	54 (78.3%)	0.21
Diabetes	8 (26.7%)	35 (28.7%)	1.00	4 (25.0%)	17 (24.6%)	1.00
Hypercholesterolemia	9 (30.0%)	27 (22.1%)	0.35	7 (43.8%)	15 (21.7%)	0.11
Smoking	17 (56.7%)	68 (55.7%)	1.00	12 (75.0%)	40 (58.0%)	0.26
Homocysteine	15.77 ± 12.86	14.21 ± 7.12	0.44	12.09 ± 3.82	15.74 ± 14.99	0.39
Days from stroke to stenting	29.50 ± 27.25	28.33 ± 30.10	0.85	25.50 ± 26.44	27.23 ± 27.85	0.82
Mori classification			0.03			0.06
Type A	2 (6.7%)	19 (15.6%)		1 (6.3%)	4 (5.8%)	
Type B	7 (23.3%)	50 (41.0%)		2 (12.5%)	29 (42.0%)	
Type C	21 (70.0%)	53 (43.4%)		13 (81.3%)	36 (52.2%)	
SR before stenting	94.90 ± 7.82	85.12 ± 9.56	<0.01	95.63 ± 6.55	84.71 ± 9.11	<0.01
SR after stenting	17.33 ± 12.01	13.32 ± 9.22	0.09	16.88 ± 13.53	12.46 ± 9.22	0.12
Months from stenting to follow-up	8.57 ± 5.07	8.46 ± 4.89	0.93	9.04 ± 5.46	8.71 ± 4.79	0.81

*The characteristics of the restenosis and non-restenosis groups were compared with the use of either an independent group t-test or a chi-square test. CTA denotes computer tomography angiography; NIHSS denotes national institute of health stroke scale; SR denotes stenosis rate*.

## Discussion

In this study, we found a rate of all peri-procedural complications of 5.8%. The restenosis rate was shown to be lower in the Neuroform group although not significantly.

Endovascular treatment of symptomatic ICAS fell into disfavor after the prematurely terminated SAMMPRIS and VISSIT trails due to significantly higher peri-procedural complications (SAMMPRIS 15%; VISSIT 24%) (3, 7). Many factors have been put forward to explain the high complication rates and many modifications of patient and stent selection were proposed to reduce the complications (8, 24, 25), but no results sufficiently convincing to change the guidelines for the prevention of secondary stroke in patients with ICAS were available (26). The prospective or retrospective cohort studies that followed modified endovascular techniques and patient selection strategies to reduce the peri-procedural complications in symptomatic ICAS patients treated by PTAS. This resulted in a much lower rate of peri-procedural complication than in SAMMPRIS (2.0–7.1 vs. 15% in SAMMPRIS) and a lower rate of stroke recurrence and death (0–9.6 vs. 10% in SAMMPRIS) during a 1-y follow-up (9–11). Our study agrees with the previous studies in finding peri-procedural complications in 5.8% of included patients and in 6.5% of matched patients. We have initiated a multi-center, randomized controlled study to further investigate whether stenting plus standard medical treatment is superior to standard medical treatment alone for symptomatic ICAS (27).

Another issue confronted in PTAS treatment was the high rate of ISR, both symptomatic and not. Restenosis was more frequent within the first 6 months after stenting (28). In the present study, the rate of ISR was higher in the Wingspan-treated group than in the Neuroform-treated group (23.3 vs. 14.3%) although not significantly. In Wingspan-treated patients, the rate of restenosis varies across studies, ranging from 10.0% to 29.7% (23, 29–31). Many reasons have been proposed to account for the ISR: (1) The radial force of the Wingspan stent is higher than that of the other intracranial stents, a parameter that has been considered the main stimulus for intimal hyperplasia (8); (2) the technique of slow inflation of a moderately undersized balloon was not included in the trial protocols, and we speculate that some restenosis could have been due to unsuspected dissections (8); (3) the Mori classification should have been taken into consideration, because a tortuous arterial access limits the apposition of the stent to the artery wall, a situation that has a tendency to encourage platelet aggregation and clot formation beneath the stent (32); (4) the length of the target lesion was positively correlated with the rate of ISR (23); (5) Post-stent dilatation may reduce symptomatic ISR (33) and (6) Younger age was a predictor of ISR in this study, which agrees with a previous study (32).

Recently, in a bid to reduce peri-operative complications and restenosis, the Enterprise stent was applied off-label to provide another option for treating patients with ICAS; the rate of peri-operative complications ranged from 4.4 to 10.0% (15, 34–37). Another study found no peri-operative complications in a consecutive cohort of 71 patients treated with the Neuroform stent (38). We speculate that our study found a higher incidence of complications compared with the prior Neuroform study for two reasons: (1) the mean length of the target lesions was greater in our sample and the stenosis degree was higher, and (2) our study included patients with artery occlusion. The Neuroform stent was used in selected patients in our study, especially when the target lesion was tortuous. Our experience indicated that the Neuroform stent could pass a tortuous artery more easily than the Wingspan, because the bending stiffness is less and the delivery of the stent is easier (16). Our study also suggested that the restenosis rate was lower in the Neuroform group, which might have been due to the lower radial force of the Neuroform stent compared with the Wingspan. However, the Neuroform stent also has shortcomings. Theoretically, the cell size of the Neuroform is larger than that of the Wingspan (16), which might increase the risk of embolism from the atherosclerotic lesion to distal branches (39), and the vessel-wall coverage is poorer with the Neuroform (16), which might increase the incidence of clot formation beneath the stent (40). However, the incidence of artery to artery embolism and the acute in-stent thrombosis did not differed significantly between the two stents in our study. More cases were needed to determine whether the subtle mechanistic differences might influence the risk of stent thrombosis and artery to artery embolism between the two stents.

This study has a few limitations. First, this was a retrospective, single-center, non-randomized study, and suffers from selection bias. Second, the sample size is relatively small for logistic regression analysis. Third, only 80.0% of our patients had been examined by CTA during follow-up, and we failed to obtain the restenosis data for the remaining cases, which could make our results very difficult to replicate. Therefore, a multicenter, prospective, controlled trial is still needed to confirm our results.

## Conclusion

This retrospective study demonstrated that the Neuroform stent was as effective and safe as the Wingspan stent in middle cerebral artery stenosis patients treated by PTAS. Younger age and higher degree of artery stenosis before stenting was found to be a predictor of ISR.

## Data Availability Statement

The original contributions presented in the study are included in the article/supplementary material, further inquiries can be directed to the corresponding author/s.

## Ethics Statement

The studies involving human participants were reviewed and approved by Medical Ethics Committee of Xinqiao Hospital, Army Medical University. The patients/participants provided their written informed consent to participate in this study.

## Author Contributions

Q-WY contributed conception and design of the study. KZ, YC, and X-HH acquisition of data. KZ performed the statistical analysis and wrote the first draft of the manuscript. Z-MQ and SL contributed guidance in statistical methods and analysis of data. Z-LG, JS, and Q-WY performed the operation in the study. All authors contributed to manuscript revision, read, and approved the submitted version.

## Conflict of Interest

The authors declare that the research was conducted in the absence of any commercial or financial relationships that could be construed as a potential conflict of interest.

## References

[1] Wang Y Zhao X Liu L Soo YO Pu Y Pan Y. Prevalence and outcomes of symptomatic intracranial large artery stenoses and occlusions in China: the Chinese Intracranial Atherosclerosis (CICAS) Study. Stroke. (2014) 45:663–9. 10.1161/STROKEAHA.113.003508 24481975

[2] Gorelick PB Wong KS Bae HJ Pandey DK. Large artery intracranial occlusive disease: a large worldwide burden but a relatively neglected frontier. Stroke. (2008) 39:2396–9. 10.1161/STROKEAHA.107.505776 18535283

[3] Chimowitz MI Lynn MJ Derdeyn CP Turan TN Fiorella D Lane BF. Stenting versus aggressive medical therapy for intracranial arterial stenosis. N Engl J Med. (2011) 365:993–1003. 10.1056/NEJMoa1105335 21899409 PMC3552515

[4] Chimowitz MI Lynn MJ Howlett-Smith H Stern BJ Hertzberg VS Frankel MR. Comparison of warfarin and aspirin for symptomatic intracranial arterial stenosis. N Engl J Med. (2005) 352:1305–16. 10.1056/NEJMoa043033 15800226

[5] Liu L Wong KS Leng X Pu Y Wang Y Jing J. Dual antiplatelet therapy in stroke and ICAS: subgroup analysis of CHANCE. Neurology. (2015) 85:1154–62. 10.1212/WNL.0000000000001972 26330567 PMC4603889

[6] Derdeyn CP Chimowitz MI Lynn MJ Fiorella D Turan TN Janis LS. Aggressive medical treatment with or without stenting in high-risk patients with intracranial artery stenosis (SAMMPRIS): the final results of a randomised trial. Lancet. (2014) 383:333–41. 10.1016/S0140-6736(13)62038-3 24168957 PMC3971471

[7] Zaidat OO Fitzsimmons BF Woodward BK Wang Z Killer-Oberpfalzer M Wakhloo A. Effect of a balloon-expandable intracranial stent vs medical therapy on risk of stroke in patients with symptomatic intracranial stenosis: the VISSIT randomized clinical trial. JAMA. (2015) 313:1240–8. 10.1001/jama.2015.1693 25803346

[8] Miao Z. Intracranial angioplasty and stenting before and after SAMMPRIS: “From Simple to Complex Strategy - The Chinese Experience”. Front Neurol. (2014) 5:129. 10.3389/fneur.2014.00129 25071714 PMC4095563

[9] Alexander MJ Zauner A Chaloupka JC Baxter B Callison RC Gupta R. WEAVE trial: final results in 152 on-label patients. Stroke. (2019) 50:889–94. 10.1161/STROKEAHA.118.023996 31125298

[10] Zhao T Zhu WY Xiong XY Li J Wang L Ding HY. Safety and efficacy of wingspan stenting for severe symptomatic atherosclerotic stenosis of the middle cerebral artery: analysis of 278 continuous cases. J Stroke Cerebrovasc Dis. (2016) 25:2368–72. 10.1016/j.jstrokecerebrovasdis.2016.05.035 27324301

[11] Wang ZL Gao BL Li TX Cai DY Zhu LF Xue JY. Outcomes of middle cerebral artery angioplasty and stenting with Wingspan at a high-volume center. Neuroradiology. (2016) 58:161–9. 10.1007/s00234-015-1611-8 26515072

[12] Gao P Wang D Zhao Z Cai Y Li T Shi H. Multicenter prospective trial of stent placement in patients with symptomatic high-grade intracranial stenosis. AJNR Am J Neuroradiol. (2016) 37:1275–80. 10.3174/ajnr.A4698 26869472 PMC7960346

[13] Salik AE Selcuk HH Zalov H Kilinc F Cirak M Kara B. Medium-term results of undersized angioplasty and stenting for symptomatic high-grade intracranial atherosclerotic stenosis with Enterprise. Int Neuroradiol. (2019) 25:484–90. 10.1177/1591019919832244 30991867 PMC6777118

[14] Natarajan SK Sonig A Mocco J Dumont TM Thind H Hartney ML. Primary stenting for acute ischemic stroke using the enterprise intracranial stent: 2-year results of a phase-I trial. J Vasc Int Neurol. (2015) 8:62–7. 26301034 PMC4535596

[15] Feng Z Duan G Zhang P Chen L Xu Y Hong B. Enterprise stent for the treatment of symptomatic intracranial atherosclerotic stenosis: an initial experience of 44 patients. BMC Neurol. (2015) 15:187. 10.1186/s12883-015-0443-9 26449986 PMC4598959

[16] Krischek O Miloslavski E Fischer S Shrivastava S Henkes H. A comparison of functional and physical properties of self-expanding intracranial stents [Neuroform3, Wingspan, Solitaire, Leo+, Enterprise]. Minim Invasive Neurosurg. (2011) 54:21–8. 10.1055/s-0031-1271681 21506064

[17] Mangubat EZ Johnson AK Keigher KM Lopes DK. Initial experience with neuroform EZ in the treatment of wide-neck cerebral aneurysms. Neurointervention. (2012) 7:34–9. 10.5469/neuroint.2012.7.1.34 22454783 PMC3299948

[18] Turk AS Niemann DB Ahmed A Aagaard-Kienitz B. Use of self-expanding stents in distal small cerebral vessels. AJNR Am J Neuroradiol. (2007) 28:533–6. 10.1007/s00234-007-0229-x 17353331 PMC7977839

[19] Chalouhi N Drueding R Starke RM Jabbour P Dumont AS Gonzalez LF. In-stent stenosis after stent-assisted coiling: incidence, predictors and clinical outcomes of 435 cases. Neurosurgery. (2013) 72:390–6. 10.1227/NEU.0b013e31828046a6 23208057

[20] Nordmeyer H Chapot R Aycil A Stracke CP Wallocha M Hadisurya MJ. Angioplasty and stenting of intracranial arterial stenosis in perforator-bearing segments: a comparison between the anterior and the posterior circulation. Front Neurol. (2018) 9:533. 10.3389/fneur.2018.00533 30038595 PMC6046376

[21] Powers WJ Rabinstein AA Ackerson T Adeoye OM Bambakidis NC Becker K. 2018 guidelines for the early management of patients with acute ischemic stroke: a Guideline for Healthcare Professionals From the American Heart Association/American Stroke Association. Stroke. (2018) 49:e46–e110. 10.1161/STR.0000000000000163 29367334

[22] Connors JJ 3rd Wojak JC. Percutaneous transluminal angioplasty for intracranial atherosclerotic lesions: evolution of technique and short-term results. J Neurosurg. (1999) 91:415–23. 10.3171/jns.1999.91.3.0415 10470816

[23] Shin YS Kim BM Suh SH Jeon P Kim DJ Kim DI. Wingspan stenting for intracranial atherosclerotic stenosis: clinical outcomes and risk factors for in-stent restenosis. Neurosurgery. (2013) 72:596–604; discussion 604. 10.1227/NEU.0b013e3182846e09 23277374

[24] Rahme RJ Aoun SG Batjer HH Bendok BR. SAMMPRIS: end of intracranial stenting for atherosclerosis or back to the drawing board? Neurosurgery. (2011) 69:N16–18. 10.1227/01.neu.0000407920.96189.cc 22067345

[25] Al-Ali F Cree T Hall S Louis S Major K Smoker S. Predictors of unfavorable outcome in intracranial angioplasty and stenting in a single-center comparison: results from the Borgess Medical Center-Intracranial Revascularization Registry. AJNR Am J Neuroradiol. (2011) 32:1221–6. 10.3174/ajnr.A2530 21546459 PMC7966065

[26] Kernan WN Ovbiagele B Black HR Bravata DM Chimowitz MI Ezekowitz MD. Guidelines for the prevention of stroke in patients with stroke and transient ischemic attack: a guideline for healthcare professionals from the American Heart Association/American Stroke Association. Stroke. (2014) 45:2160–236. 10.1161/STR.0000000000000024 24788967

[27] A Multicenter Prospective Registration Study for Stenting Plus Standard Medical Treatment Versus Standard Medical Treatment Alone for Symptomatic Intracranial Atherosclerotic Stenosis (2019).

[28] Fiorella DJ Turk AS Levy EI Pride GL Jr. Woo HH. U.S. Wingspan Registry: 12-month follow-up results. Stroke. (2011) 42:1976–81. 10.1161/STROKEAHA.111.613877 21636812

[29] Yu SC Leung TW Lee KT Hui JW Wong LK. Angioplasty and stenting of atherosclerotic middle cerebral arteries with Wingspan: evaluation of clinical outcome, restenosis, and procedure outcome. AJNR Am J Neuroradiol. (2011) 32:753–8. 10.3174/ajnr.A2363 21436335 PMC7965883

[30] Levy EI Turk AS Albuquerque FC Niemann DB Aagaard-Kienitz B Pride L. Wingspan in-stent restenosis and thrombosis: incidence, clinical presentation, and management. Neurosurgery. (2007) 61:644–50; discussion 650–641. 10.1227/01.NEU.0000290914.24976.83 17881980

[31] Costalat V Maldonado IL Vendrell JF Riquelme C Machi P Arteaga C. Endovascular treatment of symptomatic intracranial stenosis with the Wingspan stent system and Gateway PTA balloon: a multicenter series of 60 patients with acute and midterm results. J Neurosurg. (2011) 115:686–93. 10.3171/2011.5.JNS101583 21740123

[32] Turk AS Levy EI Albuquerque FC Pride GL Jr Woo H. Influence of patient age and stenosis location on wingspan in-stent restenosis. AJNR Am J Neuroradiol. (2008) 29:23–7. 10.3174/ajnr.A0869 17989366 PMC8119103

[33] Park SC Cho SH Kim MK Kim JE Jang WY Lee MK. Long-term outcome of angioplasty using a wingspan stent, post-stent balloon dilation and aggressive restenosis management for intracranial arterial stenosis. Clin Neuroradiol. (2020) 30:159–69. 10.1007/s00062-019-00793-1 31123775

[34] Lee KY Chen DY Hsu HL Chen CJ Tseng YC. Undersized angioplasty and stenting of symptomatic intracranial tight stenosis with Enterprise: evaluation of clinical and vascular outcome. Int Neuroradiol. (2016) 22:187–95. 10.1177/1591019915609165 26542728 PMC4984337

[35] Vajda Z Schmid E Guthe T Klotzsch C Lindner A Niehaus L. The modified Bose method for the endovascular treatment of intracranial atherosclerotic arterial stenoses using the Enterprise stent. Neurosurgery. (2012) 70:91–101; discussion 101. 10.1227/NEU.0b013e31822dff0f 21778921

[36] Wang X Wang Z Wang C Ji Y Ding X Zang Y. Application of the enterprise stent in atherosclerotic intracranial arterial stenosis: a series of 60 cases. Turkish Neurosurg. (2016) 26:69–76. 10.5137/1019-5149.JTN.13350-14.1 26768871

[37] Huang CM Hong YF Xing SH Xu K Xu CK Zhang WJ. Thirty-day outcomes of the enterprise stent in treating hypoperfusion of symptomatic intracranial stenosis. World Neurosurg. (2019) 129:e429–35. 10.1016/j.wneu.2019.05.167 31150855

[38] Xu H Quan T Zaidat OO Chen D Wang Z Yuan Y. Neuroform EZ stenting for symptomatic intracranial artery stenosis: 30 days outcomes in a high-volume stroke center. Front Neurol. (2019) 10:428. 10.3389/fneur.2019.00428 31156528 PMC6532552

[39] Tiekte M. Size does matter! Small-cell-stents are protection enough. J Cardiovasc Surg (Torino). (2010) 51:855–6. 21124281

[40] Ullrich H Munzel T Gori T. Coronary stent thrombosis- predictors and prevention. Dtsch Arztebl Int. (2020) 117:320–6. 10.3238/arztebl.2020.0320 32605709 PMC7358792

